# What about Your Friends? Friendship Networks and Mental Health in Critical Consciousness

**DOI:** 10.3390/youth4020056

**Published:** 2024-06-07

**Authors:** Christopher M. Wegemer, Emily Maurin-Waters, M. Alejandra Arce, Elan C. Hope, Laura Wray-Lake

**Affiliations:** 1 Department of Social Welfare, University of California, Los Angeles, CA 90095, USA; 2 Department of Psychology, University of California, Los Angeles, CA 90095, USA;; 3 Policy Research Associates, Troy, NY 12180, USA;

**Keywords:** critical consciousness, sociopolitical development, civic engagement, social networks, mental health, well-being, anxiety, depression, flourishing, friendships

## Abstract

Scholars have documented positive and negative relationships between adolescents’ critical consciousness and mental health. This study aims to clarify the role of friendship networks contributing to these associations. Using egocentric network data from a nationwide adolescent sample (*N* = 984, 55.0% female, 23.9% nonbinary, 72.7% non-white), regression analyses examined whether adolescents’ psychological distress and flourishing were predicted by their friend group’s average critical consciousness and the difference between adolescents and their friends on critical consciousness dimensions (sociopolitical action, critical agency, and critical reflection), accounting for network and demographic covariates. Higher friend group critical consciousness positively predicted flourishing, and higher friend group sociopolitical action negatively predicted psychological distress. Adolescents who participated in sociopolitical action more frequently than their friends had higher psychological distress and lower flourishing. Those with higher agency than their friends had lower flourishing. At the individual level, adolescents’ sociopolitical action predicted higher psychological distress and flourishing, critical agency predicted higher flourishing, and critical reflection predicted higher psychological distress and lower flourishing. Adolescent mental health is uniquely related to their friends’ critical consciousness. Findings highlight the utility of social network analyses for understanding social mechanisms that underlie relationships between critical consciousness and mental health.

## 1. Introduction

Participation in civic activities and the cultivation of critical consciousness are generally considered beneficial for young people’s development [1,2] and the communities they engage with [3,4]. However, amid deepening political polarization [5] and increasing racism [6], actions that push for social change against dominant power structures inherently carry risks, despite potential short- and long-term benefits. Further research is needed to understand how to reduce the risks and heighten the benefits of young people’s sociopolitical actions and beliefs that challenge social inequities, given mixed findings in the literature [7,8].

Critical consciousness, inclusive of three main dimensions of critical action, agency, and reflection, is social by nature, as each component involves thinking with, talking with, and acting with others towards shared and co-constructed social justice goals [9]. Research has suggested that adolescents can influence each other’s sociopolitical engagement through their friendships (e.g., [10–14]), and associations between youths’ activism and mental health may be moderated by relationships with peers [15,16]. Peer groups are an important developmental context and the characteristics of adolescents’ friends can contribute to a wide range of developmental processes and outcomes [17]. Accordingly, the critical consciousness of an adolescent’s friend group may shape an individual’s critical consciousness and mental health. Although the importance of relational community in critical consciousness has been highlighted in theoretical and qualitative scholarship, few studies have quantitatively explored the importance of peers. In the current study, we leverage social network concepts to examine the extent to which friends’ critical consciousness (i.e., sociopolitical action, critical agency, and critical reflection) may promote adolescents’ mental health.

### 1.1. Critical Consciousness as Collective and Communal

Adolescent social networks can be characterized in terms of overlapping friend groups [18], which provide a social context for critical consciousness development. Critical consciousness is the process of problematizing systems of oppression and acting toward justice and liberation for all oppressed people [19]. Critical consciousness includes critical action—engaging in social and political acts to reduce oppression and move society towards liberation; critical agency—believing one has the skills, knowledge, and power to engage in social and political change; and critical reflection—understanding the structural and systematic nature of oppression [1].

The interdependent evolution of action, agency, and reflection occurs in social contexts, including family, peer groups, schools, and community organizations [20,21]. Within these social contexts, critical consciousness development happens in relationships with others, through processes often involving groups, informal social interactions, and critical engagement with social structures in which youth are embedded. Sociopolitical actions are often collective and involve engaging and interacting with groups of individuals, community organizations, and social movements. Collective actions simultaneously depend on and reinforce relationships between adolescents and often include an accompanying sense of belonging and shared purpose [22]. Whether individual or collective, actions that aim to address the root causes of social issues inherently involve disrupting social relations between those experiencing injustices and those who have influence over systems of power [23]. Through praxis between action and critical reflection, youth become aware of social structures that perpetuate oppression by processing their social experiences [24], often with others in their social circles.

Critical agency involves youths’ beliefs about their competence, drive, and power to take action to pursue social change [25], each of which is cultivated relative to peers in their social circle. Empowerment theories emphasize the interdependence of agency between the individual and community levels [26,27]. Actions that challenge social injustices and foster adolescents’ agency are typically organized by groups rather than done in isolation, and accordingly, agency is socially constructed and shared. Organizing spaces consolidate youths’ collective knowledge and skills to address a particular social issue, providing opportunities for youth to feel that they can competently contribute to social change and reinforce their self-efficacy beliefs [28]. Adolescents’ motivation develops from explicit peer encouragement and emerges from spending time with friends discussing social issues and taking action [29].

Just as action and agency develop in community, critical reflection also arises from social dynamics. Critical reflection is best explored and developed within community contexts where flattened hierarchies and group discussions promote information sharing, an exploration of contrasting opinions, and critical thinking [9]. Activities and dialogue, such as storytelling and counternarratives, are common strategies for supporting shifts in perspectives about injustices and the internalization of an awareness of inequality [23,30,31]. Further, youth who collaborate with each other to address social issues often develop collective identities around shared goals [32], which can drive engagement at the group level. Collective identities are often built around shared experiences of marginalization or oppression, making the social development of critical consciousness both challenging and meaningful [33,34].

The social contexts in which youth develop critical consciousness are not benign, but are imbued with systemic oppression and struggles that complicate the difficult tasks of adopting critical perspectives and behaviors. Alongside their peers, youth confront their own identities and positionality as they seek to understand and change social conditions that have disproportionately and negatively impacted disenfranchised communities [35]. Because adolescence is a sensitive developmental period, social interactions among youth can have enduring effects on their nascent critical consciousness [36]. Shifting focus from individuals’ critical consciousness and attending to the collective nature of critical consciousness may yield important insights into the associations between critical consciousness and adolescent well-being.

### 1.2. Friendship Networks and Critical Consciousness

Drawing from social identity theory [37], youth may experience social validation and a sense of belonging that contributes to better mental health in social groups where youth have similar critical consciousness as their friends. Shared action and critical reflection build solidarity in adolescent friend groups, which can buffer against risks to mental health [16]. Further, shared civic norms among friends can facilitate adolescents’ sociopolitical actions and critical reflection [38,39], aligned with recent conceptual frameworks that highlight the importance of friendship norms in shaping prosocial adolescent behavior [40]. Barriers to entry for civic activities are likely lower when one has civically active friends [41]. The psychological costs of sociopolitical action and critical perspectives may also be lower among friend groups with high critical consciousness.

Youth may experience mental health benefits from the critical consciousness of their friend groups. Critical consciousness is associated with a sense of community and solidarity cultivated through group actions, the ability to understand and empathize with the experiences of adversity, and a sense of empowerment and control over one’s social circumstances [24,33]. Such relational attributes parallel the characteristics of high-quality friendships, which have been positively linked to mental health [42,43]. The critical consciousness of adolescent friend groups has also been linked to the development of social capital [44]. Friends high in critical consciousness may serve as positive influences by conferring socioemotional skills that youth develop through activism, such as critical thinking [23], empathy and emotion regulation [45], sense of purpose [46], and effective coping strategies [47].

Although friends’ critical consciousness may contribute positively to mental health, robust developmental research on peer socialization has shown that youth who differ from their peers may experience pressure to conform or cultivate friendships with similar peers [48,49]. Youth tend to be similar to their friends (that is, they tend to exhibit “homophily” in friend groups) on political orientation, civic behavior, and critical reflection [50–52]. Over time, adolescents may influence each other to become more similar in critical consciousness through peer pressure, modeling, mentorship, the internalization of social norms, or the expression of shared identity.

The mental health implications of differences between adolescents and their friend groups on sociopolitical action, critical agency, and critical reflection have been relatively unexplored and invoke frameworks of social comparison [53]. Political differences between youth and their friends have been linked to alienation and disruption in their relationships [54]. On the other hand, Black emerging adults report that sociopolitical similarities among friends provide a social context of support, particularly during stressful politically divisive times (e.g., national instances of police brutality; [10]). The mental health effects of dissimilarities between an adolescent and their friend groups are nuanced in two important ways.

First, the mental health effects of critical consciousness may differ for adolescents who are more critically conscious than their friend group compared to those who are less critically conscious than their friends. That is, youth who take the initiative to assert their convictions despite objections from their friends may experience more negative mental health effects than those who are unengaged while their friends advocate for change. Adolescents who are critically aware of oppression and take action on their own have reported feeling alone and vulnerable [55], which may be exacerbated if their friends do not support them or actively disagree with their stances (potentially magnifying the effects of experiences of oppression). Youth who feel alone in a social justice struggle may feel a disproportionate sense of responsibility to tackle social problems, even if they personally suffer consequences [16]. In contrast, youth with lower critical consciousness than their friends may not experience detrimental effects to their mental health. Ambivalence on political issues may not carry the same social risks as asserting a critical position [56]. Further, adolescents with low critical consciousness may enjoy advantages from the benefits conferred by their critically conscious friend group without having to participate themselves, potentially “free riding” on their friends’ engagement.

Second, the mental health effects of differences between an individual and their friend group may vary depending on the component of critical consciousness. If adolescents feel they have greater agency than their friends, the deviation from their peers may not expose them to the same social vulnerabilities as having higher critical reflection or taking action more frequently than their friends. That is, feeling more capable of causing social change than one’s friends may not result in the same type of alienation or pressure to conform as differences in sociopolitical action or critical reflection. Self-beliefs may be more private and insulated from judgments of friends, evidenced by friends’ tendency to misperceive internal attributes of their friends [57]. Also, adolescents with greater agency might feel a sense of empowerment that overrides potential social vulnerability. However, the alternative may also be true; adolescents with greater agency than their peers may feel isolated as their drive and competence extend beyond the peer group’s norms. The distinctions between friend-level mechanisms and individual-level effects of critical consciousness on mental health are unclear, as social network research remains an emerging field of study.

### 1.3. Critical Consciousness and Mental Health

Social mechanisms linking critical consciousness to mental health must be understood in the context of individual-level associations between critical consciousness and mental health. Research examining individual-level effects has emerged in recent years, but the findings have been mixed [8]. In a notable paper on youth activism and health, Ballard and Ozer [15] outline five mechanisms that connect youth sociopolitical action to health and well-being: stress and coping, empowerment, purpose and identity, social capital and connection to others, and systemic change. Critical reflection and agency may be related to mental health through these same mechanisms. Youth may confront adversity and injustice through critical consciousness as an adaptive, empowering coping strategy that uses problem-solving skills and autonomy in the face of challenges [20,58]. Ballard and Ozer [15] argue that the connections between activism and health can be promotive for some people in some circumstances, such that activism can support flourishing, psychological well-being, and better health.

In other circumstances, critical consciousness may engender psychological distress and negative emotional states as youth confront and grapple with systemic injustices [59–61]. Critical consciousness involves confronting distressing truths about the world (such as discrimination, inequality, and oppression) and pushing for social change against deeply rooted oppositional forces. This can be, and perhaps should be, troubling for young people, particularly as they become aware of the extent of injustice and develop a sense of urgency to right systemic wrongs. It is possible that adolescents who believe they can make a difference and take meaningful actions to address systemic injustices experience a boost in self-esteem and a sense of purpose and meaning in their lives that counter feelings of despair and hopelessness. On the other hand, critical consciousness is difficult work, and may expose youth to conflict or feelings of isolation and vulnerability, which can compromise mental health.

In a systematic review of 29 studies of youth critical consciousness and well-being, Maker Castro and colleagues [8] found evidence of both contentions: critical agency was generally positively associated with mental health, while the associations for action and critical reflection with mental health were both positive and negative. Critical reflection has been positively associated with indicators of positive youth development [62,63], but also associated with more depressive symptoms [64,65]. Critical agency has been associated with less anxiety and depression [66] and better self-esteem [66,67]. Sociopolitical action has been associated with better mental health [68] and less psychological distress and suicidal ideation [69], but also with worse mental health, including symptoms of depression, anxiety, and loneliness [70]. This relationship is more nuanced for some, where at the time of taking action, youth report more indicators of psychological well-being and flourishing but experience declines in well-being a year after their activism [71]. Qualitatively, youth have also reported that activism is healing and provides psychological benefits, while also being stressful and challenging to their mental health [16,72]. Importantly, recent studies have noted that the mental health effects of critical consciousness depend on the characteristics of adolescents’ particular social contexts, social identities, and relationships with peers [60,61].

### 1.4. The Current Study

The present study aims to advance understanding of social network features that relate to the mental health of civically engaged adolescents by pursuing two research goals. First, we examined the extent to which the average critical consciousness of an adolescent’s friend group relates to their mental health, after controlling for network and individual covariates. We anticipated that the average critical consciousness of adolescents’ friends would positively predict their own mental health (beyond the positive or negative effects of their own critical consciousness) due to potential social benefits that youth may gain from friends with high critical consciousness.

Second, we examined the extent to which the degree of similarity between adolescents and their friends on critical consciousness (sociopolitical action, critical agency, and critical reflection) relates to their mental health (psychological distress and flourishing). We expected that youths’ more frequent sociopolitical action and higher critical reflection than their friends would be positively related to psychological distress and negatively related to flourishing. In contrast, we expected that youths with greater critical agency than their friends would have higher flourishing and lower psychological distress, due to the protective nature of agency [64]. However, our approach is largely exploratory, as sparse literature on the topic does not support robust hypotheses.

All of our models accounted for individual-level critical consciousness, allowing us to examine the unique contributions of friend-level critical consciousness. Furthermore, all models included friend-level emotional support and structural network features of degree and density. Friends’ socioemotional support is well-known to benefit mental health and has been related to lower anxiety, depression, and feelings of isolation [73,74]. Additionally, the number of friends an adolescent has (or in network parlance, the individual’s “degree”) may offer greater access to socioemotional resources and protect against psychological distress [18,75,76]. Relatedly, a close-knit friendship structure in which many of an adolescent’s friends are also friends with each other (a “high density” network) can offer emotional safety and community [77].

## 2. Methods

### 2.1. Participants

In the spring of 2023, youth were recruited through Instagram to participate in the first wave of a planned three-year study examining the development of youth civic engagement. Prospective respondents filled out a form designed to filter out bots and scammers, and assess eligibility based on age. Specifically, we employed techniques such as image-based attention check items, the geolocation of IP addresses, the speed of survey completion, and the verification of email addresses and social media accounts, consistent with best practices [78]. Adolescents between 13 and 18 years old were invited to participate in the study. After they completed the survey, we screened responses using attention checks and inconsistencies with the initial interest form. We excluded 110 participants who indicated that they did not feel comfortable providing information about their friendship network (but we retained those who indicated that they did not have any friends). In accordance with the ethical standards of the Institutional Review Board (IRB) of the University of California, Los Angeles, parental consent was waived and we secured assent from adolescent participants. Participants were compensated $10 for completing the survey.

The final sample was 984 adolescents (*M*_age_ = 16.23) who identified as female (55.0%), nonbinary (23.9%), and male (21.1%), as well as white (27.3%), Biracial/Multiracial (23.0%), Black (17.3%), Asian (16.4%), Latine (14.2%), and other races/ethnicities (1.8%). See Table 1 for sample descriptive statistics. In addition to completing survey inventories about their own sociopolitical action, critical agency, and critical reflection, participants reported the civic characteristics of each of their friends using a single item for each of the civic constructs. All survey items are presented in Appendix A.

The missingness across study measures was less than 1% for each survey item. To account for missing data, we used the MICE (v3.16.0) package in R to conduct expectation-maximization imputation for continuous variables [79] and multinomial logit imputation for categorical variables [80] to produce a single imputed dataset through 50 iterations. Multiple imputation is generally preferable, but single imputation was empirically preferable, as network data (friend nominations and civic characteristics of friends) were not estimated. The approach was supported by Little’s MCAR test of missingness, which provided evidence that data were completely missing at random (χ^2^(2,634) = 2,710; *p* = 0.149). Participants’ indications that they did not know one or more of their friends’ civic characteristics were empirically meaningful and were left as missing. Descriptive statistics of our unstandardized study variables and network characteristics are presented in Table 2.

### 2.2. Measures

#### 2.2.1. Friendship Network

Each participant was asked to provide the first and last initials of up to six of their closest friends using a name generator item consistent with common approaches to identify egocentric networks [81]. Participants also indicated which friends were friends with each other.

#### 2.2.2. Sociopolitical Action

Participants’ sociopolitical action was measured via a 23-item scale adapted primarily from two existing scales of activism (Activism Orientation Scale, [82]; Black Community Activism Orientation Scale [83]) and from scales of sociopolitical action (Anti-Racism Action Scale [84]; Critical Consciousness Scale [85]; Youth Sociopolitical Action Scale [86]). Participants reported the frequency in which they engaged in a specific action in the past year, on a scale of 1 (*Never*), 2 (*Once*), 3 (*Sometimes*), and 4 (*Often*). Sample activities include “Campaign for a social justice cause” and “Educate others on a social or political issue”. Our items included a range of actions, including some that may not inherently challenge oppressive systems. Consequently, we used the term “sociopolitical action” to precisely represent our measure rather than “critical action”. The items demonstrated excellent internal reliability (*α* = 0.93) and were averaged to produce a single composite for sociopolitical action frequency.

In addition, we assessed participants’ sociopolitical action via a single item, “How often do you take action to address a social or political issue?” Participants responded on a scale ranging from 1 (*Never*) to 6 (*Almost every day*). Using a parallel item, participants reported the sociopolitical action of each friend they named using the same response scale, with an additional option for *I don’t know*. Respondents who indicated they did not know their friend’s frequency of sociopolitical action were marked as missing (and the missing values for these cases were not estimated), which accounted for between 7.6% and 17.5% of each of the six friend nominations. Youth reported participating a few times over the past year on average (inventory of specific actions, *M* = 2.31, *SD* = 0.63; single item of general action, *M* = 3.19, *SD* = 1.32), statistically higher than the frequency of sociopolitical action among the pool of all nominated friends (single item of general action, *M* = 2.92, *SD* = 1.45, *t* = 5.12, *p* < 0.001).

#### 2.2.3. Critical Agency

We measured the drive subscale of participants’ critical agency via four items developed for this study [25], adapted from previous scales [87,88]: “I feel determined to try to end inequalities in society”, “I feel driven by a sense of urgency to address social injustices”, “I am motivated to fight against social injustices”, and “I am compelled to participate in efforts to address injustices”. Participants evaluated the extent to which each statement was true for them on a five-point Likert scale, ranging from 1 (*Not at All True*) to 5 (*Completely True*). The items demonstrated good internal reliability (*α* = 0.85).

Participants reported on each of their friends’ critical agency with one item that paralleled the above, “They are motivated to fight against social injustices”, with the same response scale. We included an additional response option, *I don’t know*, which accounted for between 5.8% and 9.8% of each of the six friend nominations (and was coded as missing). Participants reported moderate levels of critical agency on average (*M* = 3.56, *SD* = 1.27), statistically higher than the critical agency of friends (*M* = 3.26, *SD* = 1.29, *t* = 6.26, *p* < 0.001).

#### 2.2.4. Critical Reflection

Participants’ critical reflection was assessed using three items adapted from established scales used to assess critical systems thinking: “Some people in our society benefit from unearned privileges” [89], “It is a problem that some people have more opportunities to succeed in society than others” [90], and “In our society, power is concentrated in the hands of a small number of people” [91]. A fourth item, “Many problems in our society can be attributed to systems of oppression”, was developed for this study based on previous research [65,92,93]. Participants indicated agreement with each statement using a slider scale from 0 (*Strongly Disagree*) to 100 (*Strongly Agree*). Cronbach’s alpha was acceptable (*α* = 0.70).

To assess the critical reflection of participants’ friends, participants rated the extent that they believed each friend would agree or disagree with the fourth item described above, using the same scale. We also included an additional option, *I don*’t *know*, which accounted for less than 15% of each of the six friend nominations (and was coded as missing). On average, participants reported high levels of critical reflection (*M* = 78.90, *SD* = 20.23), statistically equivalent to the critical reflection of friends (*M* = 77.69, *SD* = 19.69, *t* = 1.64, *p* = 0.100).

#### 2.2.5. Psychological Distress

Participants’ psychological distress was measured using two two-item screening tools for depression (PHQ-2; [94]) and anxiety symptoms (GAD-2; [95]), which have demonstrated acceptable sensitivity and specificity in previous research [96]. Participants reported how often they have been bothered by the following four problems: “Little interest or pleasure in doing things”, “Feeling down, depressed or hopeless”, “Feeling nervous, anxious or on edge”, and “Not being able to stop or control worrying”. Participants responded using a Likert scale ranging from 1 (*Not at all*) to 4 (*Nearly every day*). The items were averaged to create an indicator of psychological distress and the scale yielded good internal reliability (*α* = 0.85). Average scores of psychological distress indicated that participants experienced symptoms of depression and anxiety between *Several days* and *More than half the days* over the previous two weeks (*M* = 2.31, *SD* = 0.84).

#### 2.2.6. Flourishing

We used the 8-item Flourishing Scale by Diener and colleagues [97] that captured emotional well-being, resilience, and a sense of purpose. Sample items include “I lead a purposeful and meaningful life” and “I actively contribute to the happiness and well-being of others”. Items used a Likert response scale ranging from 1 (*Strongly Disagree*) to 5 (*Strongly Agree*). The items were averaged to produce an indicator of flourishing (*α* = 0.83, *M* = 3.81, *SD* = 0.62).

#### 2.2.7. Emotional Supportiveness

For each friend nominated, participants rated the emotional supportiveness of each friend, “To what extent does this friend support you emotionally?”, on a scale from 1 (*Not at all emotionally supportive*) to 5 (*Extremely emotionally supportive*). This item was adapted from Conner, Crawford, and Galioto [16]. On average, students reported that their friends were between *Somewhat emotionally supportive* and *Very emotionally supportive*, and closer to the latter (*M* = 3.81, *SD* = 0.83).

#### 2.2.8. Demographic Indicators

Demographic data included self-reported gender, race/ethnicity, age, and socioeconomic status. Gender was dummy-coded as *Male*, *Female*, and *Gender Nonbinary*/*Gender Queer*/*Other*, with *Male* as the reference group. Race/ethnicity was dummy-coded as *Biracial*/*Multiracial*, *white*, *Black*, *Latine*, *Asian*, and *Other*, with *white* as the reference group (because it was the largest category). The *Biracial*/*Multiracial* category included those who self-selected this option and those who selected more than one race/ethnicity. The *Other* category included those who self-selected it and those who selected *Arab or Middle Eastern*, *American Indian*, *Native American*, *Alaska Native*, *Indigenous*, or *Native Hawaiian or other Pacific Islander*; small sample sizes precluded further examination of these racial/ethnic categories. Age was recorded as a continuous variable between 13 and 18. Finally, a single item assessed socioeconomic status by asking participants about their family’s financial security, “Which of the following best describes your family’s financial situation?” Response categories were 1 (*We cannot buy the things we need sometimes*), 2 (*We have just enough money for the things we need*), and 3 (*We have no problem buying the things we need*). The responses were used to create a categorical variable, with the highest group as the reference.

### 2.3. Analytic Plan

In our analyses, we used egocentric network data, in which each participant (“ego”) nominated up to six friends (“alters”) and reported information about their relationships with their friends, their friends’ civic characteristics, and their friends’ relationships with each other. The resulting data provided a representation of each participant within the context of their friendship group (see [98] for more information about egocentric networks). Two structural network features were computed for each participant’s friend group. First, degree quantified the number of ties an ego has within their social network, calculated as the number of friends that each participant nominated (*M* = 3.44, *SD* = 1.96). Second, density captured the extent to which each friendship group was tightly connected, calculated as the number of actual ties divided by the total number of possible ties (ranging from 0 to 1). Higher density suggested a greater probability that participants’ close friends were also friends with each other. The average in the sample was 0.78 (*SD* = 0.21), which can be interpreted as a 78% probability of a tie existing between any two randomly chosen friends of each participant.

Next, three indicators of each friend group were calculated using the characteristics of participants and their friends. First, across each set of friends, the average of sociopolitical action, critical agency, and critical reflection were computed, respectively. Higher values indicated that each participant perceived that their friends engaged in civic activities more frequently, were more driven to engage civically, or were more likely to attribute social problems to systemic oppression (respectively). Second, the average difference between the participant and their friends was calculated for each of the three critical consciousness components by subtracting each alter’s value from the ego’s value, capturing the extent to which each participant has higher levels of an attribute than their friend group. An average difference of zero would indicate perfect homophily, that the participant and all of their friends had the same level of the construct. Positive values of average difference indicate that the participant had greater levels of the civic attribute than their friend group, whereas negative values signal that the participant had lower values relative to their friend group. We also conducted alternative analyses using the absolute value of the difference rather than the directional difference, which was less nuanced than our primary approach (see Table A1 in Appendix A). Third, the average emotional supportiveness of each set of friends was calculated by adding together the reported emotional supportiveness of each friend and dividing by the participant’s degree. Higher values indicate that a participant perceives their friend group as more emotionally supportive. We standardized all continuous variables (except degree, which is best interpreted in raw form).

Lastly, to answer our research questions, we estimated OLS regression models to predict psychological distress and flourishing using functions from R statistical software (v. 2023.06.01). Before estimating our models, we tested the primary assumptions of linear regression to verify that the data were suitable for analyses (a normal distribution of residuals, independence of observations, and homogeneity of variances). Then, we simultaneously modeled individual and friend group attributes to disaggregate the effects of friends’ critical consciousness on participants’ mental health outcomes (see [99] for an empirical elaboration of egocentric network effects). We investigated each critical consciousness component (sociopolitical action, critical agency, and critical reflection) in separate models to examine the network effects of each construct without the risk of confounding with other critical consciousness components. Each model included individual-level effects to provide a foundation for interpreting network effects; however, due to multicollinearity, the individual-level, friend-level average, and difference terms could not be included in the same model. Accordingly, two models were conducted for each construct, one estimating the effects of the friend group average, and the second estimating the effect of difference between an individual and their friend group. All models controlled for gender, age, race/ethnicity, and socioeconomic status, in addition to network features of degree, density, and the emotional supportiveness of the friend group. For all models presented, we used stepwise regression procedures to sequentially add predictors, and unless otherwise noted, the statistical significance of parameters did not change when additional predictors were included. Lastly, we conducted alternative analyses that disaggregated psychological distress into depression and anxiety components, which served as a robustness check and as a foundation for a discussion about examining multiple facets of mental health (see Tables A2 and A3 in Appendix A).

## 3. Results

### 3.1. Effects of Friends’ Average Critical Consciousness

To answer our first research question, we examined the relationships between friend-level averages of critical consciousness and mental health (psychological distress and flourishing) using correlational analyses (see Table 2). The average critical agency of participants’ respective friend groups was weakly and inversely correlated with psychological distress (*r* = −0.07, *p* = 0.036) and positively correlated with flourishing (*r* = 0.20, *p* < 0.001). Average levels of friends’ sociopolitical action were also positively correlated with flourishing (*r* = 0.16, *p* < 0.001). Friend group sociopolitical action was uncorrelated with participants’ psychological distress and friend group critical reflection was uncorrelated with both psychological distress and flourishing. For each civic construct, individual-level variables were moderately correlated with the friend group variables (sociopolitical action, *r* = 0.43, *p* < 0.001; critical agency, *r* = 0.40, *p* < 0.001; critical reflection, *r* = 0.38, *p* < 0.001).

Next, we conducted OLS regressions examining the effects of friend group averages of sociopolitical action, critical agency, and critical reflection, respectively, on psychological distress and flourishing, after accounting for individual-level civic constructs, network covariates, and demographic covariates (see Table 3). Friend-level sociopolitical action negatively predicted psychological distress, although the association was tenuous (*β* = −0.09, *p* = 0.050), and at the individual level, youths’ sociopolitical action and critical reflection were positively related to psychological distress, whereas critical agency was unrelated. Flourishing was positively predicted by all three friend-level effects (sociopolitical action, *β* = 0.12, *p* = 0.008; critical reflection, *β* = 0.12, *p* = 0.003; and critical agency, *β* = 0.16, *p* < 0.001). At the individual level, sociopolitical action and critical agency were positively associated with flourishing, although this association for critical agency became not significant when friend-level agency was included. Individual-level critical reflection was significantly negatively related to flourishing only after accounting for the positive association with friend-level critical reflection and flourishing (*β* = −0.12, *p* = 0.011).

### 3.2. Effects of Ego–Alter Differences in Critical Consciousness

Regarding our second research question, we used OLS regression models to examine the effects of average differences on sociopolitical action, critical agency, and critical reflection in turn, on psychological distress and flourishing, controlling for individual-level civic constructs, network covariates, and demographic covariates (see Table 4).

The average difference between participants and their respective friend groups on sociopolitical action positively predicted psychological distress (*β* = 0.11, *p* = 0.009), which was in the same direction as the individual-level association (*β* = 0.10, *p* = 0.023). The difference in sociopolitical action between participants and their friend groups negatively predicted flourishing (*β* = −0.08, *p* = 0.040), which diverged from the positive individual-level association between sociopolitical action and flourishing (*β* = 0.16, *p* < 0.001). That is, youths’ more frequent participation in sociopolitical action than their friends was related to greater psychological distress and lower flourishing.

The average difference between participants and their respective friend groups on critical agency negatively predicted flourishing (*β* = −0.10, *p* = 0.013), but was unassociated with psychological distress. That is, having greater critical agency than their respective friend groups was related to lower flourishing. In contrast, individual-level agency positively predicted flourishing (*β* = 0.20, *p* < 0.001). The average difference in critical reflection between participants and their friend groups was not related to psychological distress or flourishing, and individual-level critical reflection and mental health were not associated in these models.

### 3.3. Effects of Network and Demographic Covariates

Density consistently predicted greater flourishing (with standardized coefficients between 0.11 and 0.12; see Tables 3 and 4), but was not associated with psychological distress. Initial models suggested that network density predicted lower psychological distress, although this effect became nonsignificant after incorporating the average emotional support of each participant’s friend group into the models. Average emotional support negatively predicted psychological distress (with coefficients between −0.09 and −0.11) and positively predicted flourishing (with coefficients between 0.17 and 0.25). The degree of participants’ friendship networks did not predict psychological distress or flourishing (although degree was correlated with both psychological distress and individual and friend group civic constructs; see Table 2).

Gender non-binary youth had higher psychological distress (with standardized coefficients between 0.41 and 0.50) and reported lower flourishing (with standardized coefficients between −0.35 and −0.46), relative to males. Females had higher psychological distress, but the effect dissipated after including emotional support. Lower financial security predicted psychological distress relative to youth with higher financial security (with standardized coefficients between 0.28 and 0.30) but was not associated with flourishing. Consistent associations between the mental health outcomes and age and race/ethnicity were not found.

## 4. Discussion

The findings of this study suggest that social network dynamics of critical consciousness in adolescent friend groups meaningfully contribute to adolescents’ mental health. Specifically, we found that friends’ sociopolitical action, critical agency, and critical reflection were related to higher flourishing among adolescents, and friends’ sociopolitical action related to lower psychological distress, after controlling for individual-level associations between critical consciousness and mental health and other network and demographic covariates. Additionally, when adolescents were higher than their friends on sociopolitical action, they reported more psychological distress and less flourishing, and the magnitude of the effect depended on the degree of deviation from their friends. Adolescents with higher critical agency than their friends reported less flourishing. These findings suggest that social mechanisms of critical consciousness operate distinctly from individuals’ critical consciousness in shaping mental health and that friend-group norms on sociopolitical action and deviations from these norms have differing implications for adolescents’ mental health.

### 4.1. Potential Benefits of Friends’ Critical Consciousness

Friend-level and individual-level critical consciousness have different associations with adolescents’ mental health, pointing to distinct processes by which individual and group-level critical consciousness are linked to mental health. For example, adolescents’ sociopolitical action was associated with heightened psychological distress, aligning with previous research showing that youths’ critical actions are related to lower socioemotional well-being (e.g., [8,70]). In clear contrast, friend-level sociopolitical action was related to lower psychological distress, which suggests a positive role of friends’ action on individuals’ mental health. The friend-level effects were present even after accounting for the perceived supportiveness of friends. The results build on work suggesting that collective action can strengthen supportive solidarity among youth who experience oppression [33] and that sociopolitical action may cultivate beneficial socioemotional skills [45].

For critical agency, both individuals’ and friends’ critical agency related to higher flourishing, but the individual-level association dissipated after including friends’ agency. This pattern tentatively suggests that a friend group’s critical agency may be more strongly related to adolescents’ flourishing than their own personal critical agency. Collective agency has been conceptually and empirically linked to community-level health and well-being [3,22]. This finding supports scholars’ calls for more research on the collective aspects of critical consciousness, as they may have unique contributions to youth development [9].

Similar to sociopolitical action, individual-level critical reflection was related to lower flourishing, which parallels existing research suggesting that critically reflecting on systems of oppression can sometimes be detrimental to well-being [64], especially for young people who directly experience racism and other forms of oppression (e.g., [60]). At the friend level, however, critical reflection was related to greater flourishing. Being part of a social group that critically reflects on inequalities and their solutions may be a positive experience that operates in a counteractive way from an individual’s experience of critical reflection. This finding is consistent with qualitative research that shows that youth can derive support and reduce stress through critically reflective conversations with like-minded peers [10].

Notably, all three dimensions of critical consciousness at the friend level predicted more flourishing. Overall, these findings suggest that adolescents may psychologically benefit from having friends who are critically conscious. A move beyond individual-level processes is long overdue in research on critical consciousness development. Our findings suggest it is time for the field to more concertedly measure and examine collective action, reflection, and agency, as the underlying social processes may benefit adolescents’ well-being as well as development in other domains.

Several plausible explanations exist for these social network effects on adolescents’ mental health. Interacting with critically active, agentic, and reflective friends may boost adolescents’ hopefulness about the world and help them see greater meaning in life, which are aspects of flourishing. Having critically conscious friends may also lessen the depression or anxiety that can stem from understanding and experiencing the negative impacts of oppression and inequality [16]. Additionally, these average friend-level effects on adolescents’ flourishing may reflect a positive role of social norms in critical consciousness among adolescent friend groups. Research on friend-group norms during adolescence largely assumes negative influences of peers in domains such as substance use (e.g., [100]), but other literature has demonstrated that adolescents’ friends can also establish and reinforce positive norms, such as anti-racist attitudes [39] and pro-environmental values [38].

This is among the first studies to examine social network features involved in adolescents’ critical consciousness, although conceptually, peer dynamics and collective processes are understood to be crucial for adolescents’ sociopolitical development and well-being [9]. More broadly, past research has demonstrated that peers’ civic discussions and actions tend to positively predict adolescents’ sociopolitical actions [13,101], but the role of friends’ civic engagement in relation to well-being is not well understood. Our findings support the idea that adolescents’ friend groups can establish positive civic norms around taking critical actions to address social injustices, and suggest that being surrounded by friends who are taking sociopolitical action may support adolescents’ well-being.

Notably, friend-level critical consciousness was related to higher adolescent well-being even after accounting for the role of friends’ social support, which is a peer dynamic well-known to support mental health [102,103]. In other words, the effects of friend-level critical consciousness cannot be explained by adolescents feeling more supported by peers who are critically conscious. Higher friends’ sociopolitical action was correlated with individual-level sociopolitical action, and perhaps adolescents with more civically active friends are engaging in these actions with their friends. This explanation merits further examination, and perhaps the social nature of sociopolitical action offers adolescents an additional boost in meaning and fulfillment in life beyond their own individual-level actions. Friends’ sociopolitical actions may also be related to adolescents’ flourishing through giving adolescents more hope for a meaningful future inspired by their friends’ efforts. Research is open for a further exploration of social network effects in critical consciousness among friends, and our study lays an important foundation for continued study of critical action among friend and social groups and its potential benefits for individuals’ well-being. Connecting with critically conscious friends may be an important antidote to individual-level challenges to critical consciousness, an idea that merits further testing, particularly with methods that can better approximate causality.

### 4.2. Deviation from Friend-Group Civic Norms

When adolescents participated in sociopolitical action more frequently than their friends, they reported more psychological distress and lower flourishing, proportional to the degree that youth differed from their friends. Deviation from friend-group norms on sociopolitical action may take a toll on adolescents’ well-being. Theory and research on peer-group dynamics during adolescence have similarly found that deviation from peer-group norms can lead to experiences of distress, isolation, and alienation [54]. Challenging social injustices entails putting oneself at risk for psychological and social costs [55,104,105], and adolescents who take actions to challenge injustices when friends do not may be more vulnerable to the burdens of such actions. At an individual level, taking sociopolitical action predicted more psychological distress as well as more flourishing. Standing out as divergent from one’s friend group on sociopolitical actions may add to adolescents’ experiences of psychological distress and detract from their potential to flourish through sociopolitical action.

In contrast, youth who participated less frequently than their friends reported less psychological distress and more flourishing, with the magnitude of the effect increasing with greater deviation. Critical actions challenge the status quo, and youth who are not engaged may not feel pressure to conform to the actions of their friend group because it entails deviating from the broader populace. Inaction may reflect apathy rather than oppositional political perspectives [56], which may reduce the perceived significance of differences in critical consciousness with their friends. Also, it may be the case that youth experience mental health benefits from the positive attributes of their friends associated with critical consciousness, without enduring the stress of participating in actions.

Contrary to our expectations, adolescents with higher critical agency (specifically, adolescents with greater drive to address social issues) than their friends also reported lower flourishing. Scholars have previously found mental health benefits associated with civic agency [64], and the social network mechanisms involved in individuals’ and friends’ agency and their differences merit further research. It may be that youth who feel motivated and capable of causing change may experience less flourishing in the presence of friends who have lower agency because their peers may detract from their sense of resilience and purpose. Alternatively, perhaps collective agency is a more potent salve for mental health than an individual’s agency alone.

Homophily (i.e., similarity) in one’s friend group has been shown to offer adolescents a sense of social validation for their activity choices, support adolescents’ identity development, and strengthen social bonds among friend groups [106,107]. However, it should be noted that homophily on political ideology or identity characteristics is not inherently beneficial to youth. Extreme homophily may reinforce polarized beliefs about social issues or limit civic engagement [51,108], and the extent to which homophily can promote individual mental health without contributing to inter-group conflict is unclear. Scholars have increasingly documented the benefits of racial/ethnic diversity in peer groups [109], a pattern that may differ for critical consciousness. Future research may also clarify whether the magnitude of deviation matters regardless of direction. Research is greatly needed to further understand the mechanisms by which friend groups support or detract from the well-being of adolescents who challenge societal injustices.

In contrast to sociopolitical action and critical agency, differences between participants and their friends on critical reflection were not related to adolescents’ well-being. Friends’ sociopolitical actions are likely more visible to adolescents than their friends’ critical reflection or agency, which largely represent internal cognitive and motivational processes [9]. Interactions between adolescent friends tend to center on similarities in interests, and internal values or beliefs may not be as salient to friendships [107]. Thus, adolescents’ deviation from friend group norms around action may be more apparent to the adolescents and their friends than deviations on critical reflection and thus more meaningful for shaping well-being. More research is needed with other samples, longitudinal data, and other measures of well-being to replicate our findings that individual–friend differences in sociopolitical action, but not reflection, are negatively related to well-being.

Broadly, the present study supports our conjectures that distinct mechanisms may differentiate the effects of average friend-level critical consciousness from the effects attributable to deviations from the friend group. Further studies are needed to clarify the exact mechanisms responsible for the friendship effects. Social processes previously associated with well-being, such as influence, comparison, and belonging [110], could be meaningfully investigated through network analyses of critical consciousness. However, theoretical advancement will be necessary to elaborate such social processes within a critical consciousness framework. Our study suggests that the effects of friend-level critical consciousness may be meaningfully informed by theories of social capital [44], whereas the effects of adolescents’ deviations from their friends may effectively use social identity theory [37] and peer influence [49]. Conceptual synthesis will support the future examination of nuanced differences in social processes that connect sociopolitical action, critical agency, and critical reflection to various aspects of adolescent well-being.

### 4.3. Distinct Processes for Flourishing and Psychological Distress

Whereas all three dimensions of friends’ critical consciousness were related to higher adolescent flourishing, only friends’ sociopolitical action was related to lower psychological distress. Individual-level associations also showed notable divergence in patterns for psychological distress versus flourishing. Our findings underscore that adolescents’ psychological distress and flourishing are distinct components of well-being, and friends’ critical consciousness differentially predicted these two components. Mental health is multifaceted, and the absence of mental health symptomatology such as depression and anxiety does not mean that adolescents are flourishing. Flourishing captures youths’ thriving in terms of self-esteem, relationships, and sense of meaning and purpose [97,111]. It is possible and likely that adolescents can experience mental health symptoms alongside feeling fulfilled and purposeful.

The individual-level findings illustrate that adolescents’ critical consciousness, particularly sociopolitical action, and critical reflection, may be simultaneously distressing and fulfilling. These findings clarify the existing literature, which has shown mixed findings regarding whether critical consciousness benefits or detracts from adolescents’ well-being [8]. We show that both processes can be operating at the same time. Moreover, friends’ critical consciousness was more consistently related to adolescents’ flourishing than psychological distress. Adolescents may be more likely to derive meaning, purpose, and satisfaction from critical consciousness when it has a social foundation, yet depression and anxiety may be shaped by friend-level factors beyond critical consciousness, such as friends’ social support. Overall, this study shows that multiple dimensions of mental health should be considered alongside critical consciousness at the individual and group level.

Lastly, characteristics of the macro-level social contexts in which friend groups are embedded (e.g., the sociopolitical environment of their school or neighborhood) may shape both the friend-level and individual-level effects of critical consciousness on mental health. Although friend-level effects are distinct from individual-level effects, both are shaped by the broader sociopolitical climate, and the particular manifestations and distinctions of friend-level processes may vary across locations and cultures. Relatedly, shared social environment and identity can promote homophily among youth on both critical consciousness and mental health, as friends who have similarities in sociopolitical action and critical reflection are likely to experience the same pressures and incentives from their shared community environment and culture. Future research may examine the extent to which contextual factors (such as political homophily in a school, or disciplinary policies that exacerbate social marginalization) may modulate the relationship between critical consciousness and mental health outcomes. Importantly, as highlighted in this special issue, associations between critical consciousness and mental health may differ for marginalized youth who are the targets of oppression firsthand. Research is needed to examine whether friendship networks function differently across race/ethnicity, gender, sexual orientation, and other social identity characteristics in the context of critical consciousness and mental health.

### 4.4. Limitations

A primary limitation of this study is its cross-sectional design, which limits our understanding of the processes by which social network dynamics in critical consciousness relate to mental health. The influence of friends likely unfolds over time, and friend groups may change both in their composition and in the level of their critical consciousness. Our study cannot capture the ongoing growth and change in adolescents’ social networks, nor can we determine whether associations between friends’ critical consciousness and adolescents’ well-being change in strength or direction over time. Longitudinal network techniques such as stochastic actor-based modeling (see [14]) could meaningfully disaggregate the effects of peer influence, friend selection, and the critical consciousness of friendships (dyadic and friend group) from individual-level critical consciousness, as well as identify potential individual-level by friend-level interactions.

Relatedly, we could not capture the directionality of effects, and although, based on theory and evidence, we have framed the study in terms of critical consciousness predicting mental health, the reverse direction of effects cannot be ruled out. For example, adolescents with heightened flourishing may seek out more critically conscious friend groups, as having purpose may translate into greater interest in friends who reflect on and challenge inequalities in their community and society. Furthermore, adolescents who experience more psychological distress may seek critical action as a coping mechanism [58], and youth with lower flourishing may engage in critical reflection as part of seeking meaning. Longitudinal models can clarify the directionality of effects to some extent. Quasi-experimental approaches such as propensity score modeling may be especially useful in assessing the potential benefits of friends’ critical consciousness for adolescents’ well-being.

Another limitation is that we used regression models rather than social network methodologies. We made this choice to address our research questions more simply and directly, but future analyses will utilize Krivitsky and Morris’s [112] novel application of pseudo-exponential random graph modeling to examine how critical consciousness may predict friendship ties. Also, our egocentric network data only solicits adolescents’ assessments of their respective close friends and does not provide complete network data. Although youth are accurate in gauging the frequency of their peers’ sociopolitical actions [38], adolescents’ evaluation of others’ critical consciousness may be subject to uncertainty, as is evident from the 10–20% of youth who indicated they did not know their friends’ civic attributes. The field of critical consciousness would benefit from more studies that collect “complete” network data, which specifies ties between all individuals within a particular network. Such data not only provides a more accurate assessment of relationships, but can support longitudinal network methods (e.g., stochastic actor-based modeling, see [113]) that are capable of clarifying the extent to which an adolescent’s changes in mental health are attributable to the influence of peer mental health, friendship network changes, or changes in the critical consciousness of the adolescent or their friends. Lastly, our data only contained friendship nominations, but other information about the friendships would enable a more nuanced evaluation of the network effects. Information about co-participation in sociopolitical actions or co-membership in civic groups may elucidate particular friendship mechanisms that contribute to positive mental health outcomes.

### 4.5. Implications and Conclusion

This study advances the theory and practice of critical consciousness by showcasing the value of considering peers’ critical consciousness and its benefits for mental health. Our study also demonstrates the potential negative mental health consequences of being active, agentic, or reflective without a like-minded friend group, and points to the critical importance of better understanding the social conditions under which youth may experience negative mental health consequences as a result of their sociopolitical action. By examining the social conditions that exacerbate or buffer the mental health risks of critical consciousness, we can better structure environments and opportunities that support youths’ civic engagement and their mental health and well-being. Social network analysis will be an invaluable tool in this endeavor. Our findings validate and support the existing practices of organizations and youth-led social movements that focus on building social bonds and engaging in collective action.

## Figures and Tables

**Table 1.** Demographics of sample.

		*N*	%
Age			
	13	5	(0.5%)
	14	55	(5.6%)
	15	179	(18.2%)
	16	292	(29.7%)
	17	375	(38.1%)
	18	78	(7.9%)
Race/ethnicity			
	Biracial/Multiracial	226	(23.0%)
	White	268	(27.3%)
	Black	170	(17.3%)
	Latine	139	(14.2%)
	Asian	161	(16.4%)
	Other	18	(1.8%)
Gender Identity			
	Woman	539	(55.0%)
	Man	207	(21.1%)
	Gender Nonbinary, Gender Queer, etc.	234	(23.9%)
Family financial insecurity			
	We cannot buy the things we need sometimes	149	(15.2%)
	We have just enough money for the things we need	443	(45.2%)
	We have no problem buying the things we need	389	(39.7%)

Note. *N* = 984. Categories were mutually exclusive. Any participants who selected more than one race/ethnicity were included in the Bi/Multiracial category. “Other” racial/ethnic category includes participants who selected “Other”, as well as those who selected “Arab or Middle Eastern”, “American Indian, Native American, Alaska Native, Indigenous”, or “Native Hawaiian or other Pacific Islander”.

**Table 2.** Correlations between study variables.

		1	2	3	4	5	6	7	8	9	10	11	12	13	14
1	Psychological distress	1													
2	Flourishing	−0.41 ***	1												
3	Sociopolitical action	0.17 ***	0.08 **	1											
4	Critical agency	0.05	0.16 ***	0.44 ***	1										
5	Critical reflection	0.18 ***	−0.11 ***	0.27 ***	0.29 ***	1									
6	Average sociopolitical action of friend group	−0.01	0.16 ***	0.43 ***	0.26 ***	0.06	1								
7	Average critical agency of friend group	−0.07 *	0.20 ***	0.34 ***	0.40 ***	0.12 ***	0.65 ***	1							
8	Average critical reflection of friend group	0.05	0.04	0.17 ***	0.23 ***	0.38 ***	0.25 ***	0.32 ***	1						
9	Difference between ego and alters on sociopolitical action	0.12 ***	−0.04	0.24 ***	0.13 ***	0.12 ***	−0.49 ***	−0.31 ***	−0.08 *	1					
10	Difference between ego and alters on critical agency	0.10 **	−0.06	0.06	0.49 ***	0.15 ***	−0.29 ***	−0.46 ***	−0.08 *	0.36 ***	1				
11	Difference between ego and alters on critical reflection	0.10 **	−0.07 *	0.08 *	0.11 ***	0.37 ***	−0.09 *	−0.11 ***	−0.45 ***	0.15 ***	0.23 ***	1			
12	Degree	0.07 *	0.01	0.07 *	0.07 *	0.15 ***	−0.08 *	−0.07 *	0.15 ***	0.08 *	0.13 ***	0.05	1		
13	Density of friend group	−0.11 ***	0.09 **	−0.02	−0.02	−0.11 ***	0.10 **	0.11 **	−0.10 **	−0.07 *	−0.12 ***	−0.02	−0.62 ***	1	
14	Average emotional support of friend group	−0.11 **	0.25 ***	0.09 *	0.20 ***	0.04	0.22 ***	0.33 ***	0.14 ***	−0.06	−0.15 ***	−0.10 *	−0.18 ***	0.14 ***	1
Mean		2.31	3.81	2.31	3.48	80.78	3.02	3.30	74.50	0.24	0.30	4.27	3.44	0.78	3.81
S.D.		0.84	0.61	0.63	1.02	15.25	1.28	1.02	19.42	1.31	1.34	20.06	1.97	0.21	0.83
Min		1	1	1	1	0	1	1	0	−6	−4	−100	0	0	1
Max		4	5	4	5	100	7	5	100	6	4	100	6	1	5

* *p* < 0.05;

** *p* < 0.01;

*** *p* < 0.001.

**Table 3.** Regression models, effects of individual and friend group critical consciousness on psychological distress and flourishing.

	Psychological Distress			Flourishing		
	Sociopolitical Action	Critical Agency	Critical Reflection	Sociopolitical Action	Critical Agency	Critical Reflection
Participant sociopolitical action	0.17 *** (0.049)			0.09 * (0.046)		
Average sociopolitical action of friend group	−0.09 * (0.046)			0.12 ** (0.043)		
Participant critical agency		0.04 (0.046)			0.08 (0.044)	
Average critical agency of friend group		−0.08 (0.044)			0.16 *** (0.042)	
Participant critical reflection			0.12 * (0.047)			−0.12 * (0.045)
Average critical reflection of friend group			−0.03 (0.041)			0.12 ** (0.039)
Degree	−0.03 (0.049)	−0.03 (0.048)	−0.03 (0.048)	0.02 (0.046)	0.02 (0.045)	0.05 (0.047)
Density of friend group	−0.09 (0.059)	−0.09 (0.057)	−0.05 (0.059)	0.13 * (0.055)	0.12 * (0.055)	0.12 * (0.057)
Average emotional support of friend group	−0.10 * (0.044)	−0.09 * (0.044)	−0.11 * (0.043)	0.17 *** (0.041)	0.16 *** (0.042)	0.22 *** (0.041)
Constant	−0.64 (0.626)	−0.59 (0.615)	−0.60 (0.624)	1.10 (0.587)	0.90 (0.584)	1.09 (0.603)
Observations	555	577	568	555	577	568
Adjusted R^2^	0.068	0.059	0.065	0.118	0.135	0.121
Residual Std. Error	0.959	0.958	0.953	0.900	0.910	0.920
F Statistic	3.910 *** (df = 14; 540)	3.594 *** (df = 14; 562)	3.817 *** (df = 14; 553)	6.279 *** (df = 14; 540)	7.400 *** (df = 14; 562)	6.564 *** (df = 14; 553)

Note. All continuous variables except degree were standardized, including the outcomes. Standard errors are shown in parentheses. All models included controls for gender, age, race/ethnicity, and SES. Psychological distress is measured as anxiety and depression. The scale average was used for participant sociopolitical action, critical agency, and critical reflection. The single item was used for the average sociopolitical action, critical agency, and critical reflection of the friend group.

* *p* < 0.05;

** *p* < 0.01;

*** *p* < 0.001.

**Table 4.** Regression models, effects of difference between ego and alter critical consciousness on psychological distress and flourishing.

	Psychological Distress			Flourishing		
	Sociopolitical Action	Critical Agency	Critical Reflection	Sociopolitical Action	Critical Agency	Critical Reflection
Participant sociopolitical action	0.10 * (0.046)			0.16 *** (0.043)		
Difference between ego and alters on sociopolitical action	0.11 ** (0.043)			−0.08 * (0.041)		
Participant critical agency		−0.02 (0.049)			0.20 *** (0.047)	
Difference between ego and alters on critical agency		0.06 (0.044)			−0.10 * (0.042)	
Participant critical reflection			0.08 (0.050)			−0.06 (0.048)
Difference between ego and alters on critical reflection			0.05 (0.042)			−0.02 (0.041)
Degree	−0.03 (0.049)	−0.02 (0.048)	−0.02 (0.049)	0.02 (0.046)	0.02 (0.046)	0.04 (0.046)
Density of friend group	−0.09 (0.059)	−0.09 (0.058)	−0.05 (0.059)	0.13 * (0.056)	0.12 * (0.055)	0.12 * (0.056)
Average emotional support of friend group	−0.10 * (0.043)	−0.10 * (0.044)	−0.11 ** (0.043)	0.18 *** (0.041)	0.17 *** (0.042)	0.25 *** (0.041)
Constant	−0.43 (0.631)	−0.46 (0.627)	−0.38 (0.638)	0.97 (0.595)	0.69 (0.599)	0.86 (0.609)
Observations	555	577	556	555	577	556
Adjusted R^2^	0.074	0.057	0.062	0.113	0.123	0.122
Residual Std. Error	0.957	0.959	0.949	0.902	0.916	0.906
F Statistic	4.148 *** (df = 14; 540)	3.501 *** (df = 14; 562)	3.640 *** (df = 14; 541)	6.046 *** (df = 14; 540)	6.767 *** (df = 14; 562)	6.159 *** (df = 15; 540)

Note. All continuous variables except degree were standardized, including the outcome. Standard errors are shown in parentheses. All models included controls for gender, age, race/ethnicity, and SES. Psychological distress is measured as anxiety and depression. The scale average was used for participant sociopolitical action, critical agency, and critical reflection. The single item for all three measures of critical consciousness was used for the difference between ego and alters. The difference between ego and alters is measured as the average difference between the participant and each of their friends. That is, the difference will have positive values if the participant is higher than their friends on the attribute, whereas the difference will have negative values if the participant is lower than their friends on the attribute. Consequently, positive coefficients for the term indicate that the extent to which a participant has a higher level of the particular critical consciousness component than their friends is related to higher values of the outcome.

* *p* < 0.05;

** *p* < 0.01;

*** *p* < 0.001.

## Data Availability

The data supporting the conclusions of this article will be made available by the authors on request.

## Appendix A. Survey Items

### Appendix A.1. Friendship Network

Adapted from:

Marsden, P. V. (2011). Survey methods for network data. *The SAGE Handbook of Social Network Analysis*, *25*, 370–388.

1. Think about your closest friends. Write the first name and last initial of each of your friends on the lines below, starting with your closest friend first. A new line will appear after each friend that you enter and you can list up to six friends. Remember, your responses will be kept confidential. Please complete this question as well as you can.
  [Six open-ended lines, with one appearing at a time to prevent line skipping]
  [Display only for participants who don’t nominate any friends]
2. You did not enter any friends in the previous question. Please select the response that is most accurate for you:
  I don’t feel comfortable entering my friends’ initials
  I don’t have any friends to enter
  Other reason: [Open-ended]
3. Is [friend’s name] also friends with any of your other friends below? Select all that apply.
  [Repeat for all combinations of their friends]
  For the following questions, please think about [first friend’s name].
  [Repeat the next 5 items for each friend]

### Appendix A.2. Sociopolitical Action

#### 23-item measure

Adapted from:

Aldana, A., Bañales, J., & Richards-Schuster, K. (2019). Youth anti-racist engagement: Conceptualization, development, and validation of an anti-racism action scale. *Adolescent Research Review*, *4*, 369–381.

Corning, A. F., & Myers, D. J. (2002). Individual orientation toward engagement in social action. *Political Psychology*, *23*(4), 703–729.

Diemer, M. A., Rapa, L. J., Park, C. J., & Perry, J. C. (2017). Development and validation of the critical consciousness scale. Youth & Society, 49(4), 461–483.

Hope, E. C., Pender, K. N., & Riddick, K. N. (2019). Development and validation of the Black community activism orientation scale. *Journal of Black Psychology*, *45*(3), 185–214.

Wilf, S., & Wray-Lake, L. (2023). Development and Validation of the Youth Sociopolitical Action Scale for Social Media (SASSM). *Adolescent Research Review*, 1–14.

1. Now we’re going to ask you about a lot of specific activities. How often did you engage in the following actions IN THE PAST YEAR? These actions could be done at school or out of school, and could be done in person or online.
  [Response options: Never, Once, Sometimes, Often]
  1. Organize a political or social issue-related event (e.g., talk, support group, protest)
  2. Serve in a leadership role in a political or social issue-related organization
  3. Attend a meeting or event for a political or social issue
  4. Campaign for a political candidate
  5. Campaign for a social justice cause
  6. Send a letter or email about a political or social issue to a person in a position of power (e.g., politician, company manager, or school administrator)
  7. Speak at a meeting or hearing to advocate for or against a policy
  8. Attend a protest, rally, march, or demonstration
  9. Engage in a political activity in which you suspected there would be a confrontation with the police or possible arrest
  10. Engage in a political activity in which you feared for your personal safety
  11. Post on social media to advocate for a policy change.
  12. Go out of your way to learn about a political or social issue
  13. Sign a petition for a political or social cause
  14. Distribute information about a social or political cause
  15. Boycott a brand, platform, or product for political or social issue-related reasons
  16. Try to change someone’s mind about a social or political issue
  17. Present facts to contest another person’s social or political statement
  18. Confront or check someone who made an inappropriate statement or joke that was prejudiced
  19. Have a difficult conversation with someone about politics or a social justice issue
  20. Challenge stigmas, stereotypes, or prejudices on social media
  21. Call out injustice to hold individuals or institutions accountable for their actions
  22. Educate others on a social or political issue
  23. Encourage others to take action on a social or political issue

#### Single-item indicator

Asked of participants:

1. How often do you take action to address a social or political issue?
  Never
  Less than once a year
  A few times a year
  A few times a month
  At least once a week
  Almost everyday
  Asked of participants about each of the friends they nominated:
2. How often does [friend’s name] take action to address a social or political issue?
  Never
  Less than once a year
  A few times a year
  A few times a month
  At least once a week
  Almost everyday
  I don’t know

### Appendix A.3. Critical Agency

Adapted from:

Diemer, M. A., Frisby, M. B., Pinedo, A., Bardelli, E., Elliot, E., Harris, E., McAlister, S., & Voight, A. M. (2022). Development of the short critical consciousness scale (ShoCCS). *Applied Developmental Science*, *26*(3), 409–425.

McWhirter, E. H., & McWhirter, B. T. (2016). Critical consciousness and vocational development among Latina/o high school youth: Initial development and testing of a measure. *Journal of Career Assessment*, *24*(3), 543–558.

#### 4-item measure asked of participants:

1. How true are each of the following statements for you?
  [1—Not at all true, 2—A little bit true, 3—Somewhat true, 4—Mostly true, 5—Completely true]
  1. I feel determined to try to end inequalities in society.
  2. I feel driven by a sense of urgency to address social injustices.
  3. I am motivated to fight against social injustices.
  4. I am compelled to participate in efforts to address injustices.

#### Single item asked of participants about each of the friends they nominated:

1. To what extent do you think the following statement is true about [friend’s name]?
  They are motivated to fight against social injustices.
  Not at all true
  little bit true
  Somewhat true
  Mostly true
  Completely true
  don’t know

### Appendix A.4. Critical Reflection

Adapted from:

Godfrey, E. B., Burson, E. L., Yanisch, T. M., Hughes, D., & Way, N. (2019). A bitter pill to swallow? Patterns of critical consciousness and socioemotional and academic well-being in early adolescence. *Developmental Psychology*, *55*(3), 525.

Neville, H. A., Lilly, R. L, Duran, G., Lee, R. M., Browne, L. (2000). Construction and initial validation of the Color-Blind Racial Attitudes Scale (CoBRAS). *Journal of Counseling Psychology*, *47*, 59–70.

Pratto, F., Sidanius, J., Stallworth, L. M., & Malle, B. F. (1994). Social dominance orientation: A personality variable predicting social and political attitudes. *Journal of Personality and Social Psychology*, *67*(4), 741.

Shin, R. Q., Ezeofor, I., Smith, L. C., Welch, J. C., & Goodrich, K. M. (2016). The development and validation of the Contemporary Critical Consciousness Measure. *Journal of Counseling Psychology*, *63*(2), 210.

Watts, R. J., & Halkovic, A. (2022). Sociopolitical development and social identities. *Journal of Research on Adolescence*, *32*(4), 1270–1279.

Windsor, L. C., Jemal, A., Goffnett, J., Smith, D. C., & Sarol Jr, J. (2022). Linking critical consciousness and health: The utility of the critical reflection about social determinants of health scale (CR_SDH). *SSM-population health*, *17*, 101034.

#### 4-item measure asked of participants:

1. How much do you agree or disagree with the following statements?
  [0 strongly disagree to 100 strongly agree]
  1. Some people in our society benefit from unearned privileges.
  2. It is a problem that some people have more opportunities to succeed in society than others.
  3. In our society, power is concentrated in the hands of a small number of people.
  4. Many problems in our society can be attributed to systems of oppression.

#### Single item asked of participants about each of the friends they nominated:

1. To what extent do you think that [friend’s name] would agree or disagree with the following statement?
  Many problems in our society can be attributed to systems of oppression.
  [0 strongly disagree to 100 strongly agree]

### Appendix A.5. Psychological Distress

Adapted from:

Kroenke, K., Spitzer, R. L., & Williams, J. B. W. (2003). The Patient Health Questionnaire-2: Validity of a two-item depression screener. *Medical Care*, *41*(11), 1284–1292. https://doi.org/10.1097/01.MLR.0000093487.78664.3C

Kroenke, K., Spitzer, R. L., Williams, J. B. W., Monahan, P. O., & Löwe, B. (2007). Anxiety disorders in primary care: Prevalence, impairment, comorbidity, and detection. *Annals of Internal Medicine*, *146*(5), 317–325.

Staples, L. G., Dear, B. F., Gandy, M., Fogliati, V., Fogliati, R., Karin, E., Nielssen, O., & Titov, N. (2019). Psychometric properties and clinical utility of brief measures of depression, anxiety, and general distress: The PHQ-2, GAD-2, and K-6. General hospital psychiatry, 56, 13–18.

1. Over the past 2 weeks, how often have you been bothered by the following problems?
  [Not at all, Several days, More than half the days, Nearly every day]
  1. Little interest or pleasure in doing things
  2. Feeling down, depressed or hopeless
  3. Feeling nervous, anxious or on edge
  4. Not being able to stop or control worrying

### Appendix A.6. Flourishing

Adapted from:

Diener, E., Wirtz, D., Tov, W., Kim-Prieto, C., Choi, D. W., Oishi, S., & Biswas-Diener, R. (2010). New well-being measures: Short scales to assess flourishing and positive and negative feelings. *Social indicators research*, *97*, 143–156.

1. Please rate your level of agreement with the following statements.
  [Strongly disagree, Disagree, Neither agree nor disagree, Agree, Strongly agree]
  1. I lead a purposeful and meaningful life.
  2. People respect me.
  3. I am optimistic about my future.
  4. I am a good person and live a good life.
  5. I am competent and capable in the activities that are important to me.
  6. I actively contribute to the happiness and well-being of others.
  7. I am engaged and interested in my daily activities.
  8. My social relationships are supportive and rewarding.

### Appendix A.7. Emotional Supportiveness

Adapted from:

Conner, J. O., Crawford, E., & Galioto, M. (2023). The mental health effects of student activism: Persisting despite psychological costs. *Journal of Adolescent Research*, *38*(1), 80–109.

#### Asked of participants about each of the friends they nominated:

1. To what extent does this friend support you emotionally?
  Not at all emotionally supportive
  A little emotionally supportive
  Somewhat emotionally supportive
  Very emotionally supportive
  Extremely emotionally supportive

### Appendix A.8. Demographic Indicators

#### Gender

1. Please select the answer(s) that best describes you. Please choose all that apply:
  I identify as genderqueer, gender non-binary, gender fluid, or two-spirited
  I identify as transgender
  I identify with the gender assigned to me at birth (cisgender)
  Other (open-ended)
2. Please select the answer(s) that best describes you. Please choose all that apply:
  I identify as genderqueer, gender non-binary, gender fluid, or two-spirited
  I identify as transgender
  I identify with the gender assigned to me at birth (cisgender)
  I identify as something else (open-ended)
3. I identify as:
  Please choose all that apply:
  Two-Spirited
  Non-Binary
  Gender Fluid
  Genderqueer
  Male
  Female
  Gender Questioning
  An identity not listed [Open-ended]*

#### Race/ethnicity

1. I identify with the following race or ethnic groups: (Please choose all that apply).
  Biracial or Multiracial
  White or Caucasian
  Native Hawaiian or other Pacific Islander
  Latino/a/x
  Asian including South Asian
  Arab or Middle Eastern
  American Indian, Native American, Alaska Native, Indigenous
  Black/African American

#### Age

1. How old are you?
  Under 13
  13
  14
  15
  16
  17
  18
  19
  20
  21 or older

#### Socioeconomic status

1. Which of the following best describes your family’s financial situation?
  We cannot buy the things we need sometimes
  We have just enough money for the things we need.
  We have no problem buying the things we need.

**Table A1.** Regression models, effects of absolute value of difference between ego and alter critical consciousness on psychological distress and flourishing.

	Psychological Distress			Flourishing		
	Sociopolitical Action	Critical Agency	Critical Reflection	Sociopolitical Action	Critical Agency	Critical Reflection
Participant sociopolitical action	0.12 ** (0.045)			0.16 *** (0.042)		
Difference between ego and alters on sociopolitical action	0.14 * (0.060)			−0.18 ** (0.056)		
Participant critical agency		−0.01 (0.043)			0.14 *** (0.047)	
Difference between ego and alters on critical agency		0.06 (0.057)			−0.10 (0.042)	
Participant critical reflection			0.10 * (0.046)			−0.07 (0.044)
Difference between ego and alters on critical reflection			−0.09 (0.053)			−0.0003 (0.051)
Degree	−0.01 (0.049)	−0.02 (0.048)	−0.02 (0.049)	0.002 (0.046)	0.017 (0.046)	0.04 (0.046)
Density of friend group	−0.09 (0.059)	−0.10 (0.057)	−0.05 (0.058)	0.13 * (0.055)	0.13 * (0.055)	0.12 * (0.056)
Average emotional support of friend group	−0.11 * (0.043)	−0.11 * (0.043)	−0.13 ** (0.043)	0.18 *** (0.041)	0.19 *** (0.042)	0.25 *** (0.041)
Constant	−0.41 (0.635)	−0.49 (0.630)	−0.78 (0.650)	0.82 (0.595)	0.74 (0.603)	0.91 (0.622)
Observations	555	577	556	555	577	556
Adjusted R^2^	0.072	0.056	0.065	0.123	0.118	0.120
Residual Std. Error	0.958	0.959	0.948	0.897	0.918	0.907
F Statistic	4.056 *** (df = 14; 540)	3.440 *** (df = 14; 562)	3.771 *** (df = 14; 541)	6.538 *** (df = 14; 540)	6.524 *** (df = 14; 562)	6.422 *** (df = 15; 541)

Note. All continuous variables except degree were standardized, including the outcome. Standard errors are shown in parentheses. All models included controls for gender, age, race/ethnicity, and SES. Psychological distress is measured as anxiety and depression. The scale average was used for participants’ sociopolitical action, critical agency, and critical reflection. For each component of critical consciousness, the single item was used to calculate the absolute value of the difference between each participant and their respective friends. That is, the difference will have higher positive values that measure the extent to which participants deviate from their friends on each critical consciousness component, regardless of whether the participants have a higher or lower value than their friends. Consequently, positive coefficients for the term indicate that the extent to which a participant has a higher level of the particular critical consciousness component than their friends is related to higher values of the outcome.

* *p* < 0.05;

** *p* < 0.01;

*** *p* < 0.001.

**Table A2.** Regression models, effects of individual and friend group critical consciousness on anxiety and depression.

	Anxiety			Depression		
	Sociopolitical Action	Critical Agency	Critical Reflection	Sociopolitical Action	Critical Agency	Critical Reflection
Participant sociopolitical action	0.19 *** (0.05)			0.11 * (0.05)		
Average sociopolitical action of friend group	−0.12 ** (0.046)			−0.04 (0.046)		
Participant critical agency		0.06 (0.046)			0.01 (0.046)	
Average critical agency of friend group		−0.08 (0.044)			−0.06 (0.044)	
Participant critical reflection			0.10 * (0.047)			0.11 * (0.047)
Average critical reflection of friend group			−0.01 (0.041)			−0.05 (0.041)
Degree	0.02 (0.049)	0.03 (0.048)	0.03 (0.049)	−0.07 (0.049)	−0.08 (0.048)	−0.09 * (0.048)
Density of friend group	−0.07 (0.059)	−0.08 (0.058)	−0.03 (0.059)	−0.09 (0.059)	−0.09 (0.057)	−0.06 (0.059)
Average emotional support of friend group	−0.04 (0.044)	−0.04 (0.044)	−0.05 (0.043)	−0.14 *** (0.044)	−0.13 ** (0.044)	−0.15 *** (0.043)
Gender (male is reference)						
Female	0.10 (0.102)	0.08 (0.101)	0.14 (0.104)	0.05 (0.102)	0.05 (0.101)	0.09 (0.103)
Nonbinary	0.39 ** (0.127)	0.40 ** (0.124)	0.45 *** (0.127)	0.38 ** (0.127)	0.40 ** (0.124)	0.46 *** (0.127)
Age	0.01 (0.039)	0.01 (0.038)	0.02 (0.038)	0.03 (0.039)	0.02 (0.038)	0.01 (0.038)
Race/ethnicity (white is reference)						
Bi/Multiracial	0.21 (0.114)	0.31 ** (0.113)	0.26 * (0.115)	0.07 (0.114)	0.10 (0.113)	0.05 (0.115)
Latine	0.10 (0.141)	0.08 (0.138)	0.04 (0.14)	0.03 (0.142)	0.02 (0.137)	−0.01 (0.139)
Asian	0.06 (0.133)	0.05 (0.128)	0.08 (0.129)	0.14 (0.134)	0.08 (0.128)	0.13 (0.128)
Black	0.06 (0.128)	0.02 (0.125)	0.02 (0.124)	−0.12 (0.128)	−0.16 (0.125)	−0.12 (0.123)
Family socioeconomic status (High SES is reference)						
Low SES	0.226 (0.132)	0.25 (0.13)	0.24 (0.129)	0.28 * (0.133)	0.29 * (0.129)	0.26 * (0.129)
Med SES	0.086 (0.092)	0.06 (0.09)	0.05 (0.092)	0.18 (0.092)	0.15 (0.09)	0.18 * (0.091)
Constant	−0.624 (0.628)	−0.61 (0.617)	−0.75 (0.629)	−0.53 (0.628)	−0.46 (0.615)	−0.33 (0.626)
Observations	555	577	568	555	577	568
Adjusted R^2^	0.063	0.051	0.053	0.056	0.053	0.063
Residual Std. Error	0.962	0.961	0.960	0.963	0.958	0.955
F Statistic	3.654 *** (df = 14; 540)	3.233 *** (df = 14; 562)	3.271 *** (df = 14; 553)	3.358 *** (df = 14; 540)	3.313 *** (df = 14; 562)	3.709 *** (df = 14; 553)

Note. All continuous variables except degree were standardized, including the outcome. Standard errors are shown in parentheses. All models included controls for gender, age, race/ethnicity, and SES. The scale average was used for participant sociopolitical action, critical agency, and critical reflection. The single item was used for the average sociopolitical action, critical agency, and critical reflection of the friend group.

* *p* < 0.05;

** *p* < 0.01;

*** *p* < 0.001.

**Table A3.** Regression models, effects of difference between ego and alter critical consciousness on anxiety and depression.

	Anxiety			Depression		
	Sociopolitical Action	Critical Agency	Critical Reflection	Sociopolitical Action	Critical Agency	Critical Reflection
Participant sociopolitical action	0.11 * (0.046)			0.08 (0.046)		
Difference between ego and alters on sociopolitical action	0.12 ** (0.043)			0.09 * (0.043)		
Participant critical agency		0.00 (0.049)			−0.04 (0.049)	
Difference between ego and alters on critical agency		0.06 (0.044)			0.05 (0.044)	
Participant critical reflection			0.08 (0.050)			0.06 (0.050)
Difference between ego and alters on critical reflection			0.02 (0.043)			0.06 (0.043)
Degree	0.02 (0.049)	0.03 (0.048)	0.04 (0.049)	−0.07 (0.049)	−0.08 (0.048)	−0.07 (0.049)
Density of friend group	−0.08 (0.059)	−0.08 (0.058)	−0.03 (0.059)	−0.09 (0.059)	−0.09 (0.058)	−0.07 (0.059)
Average emotional support of friend group	−0.05 (0.044)	−0.05 (0.044)	−0.05 (0.043)	−0.14 ** (0.044)	−0.13 ** (0.044)	−0.16 *** (0.043)
Gender (male is reference)						
Female	0.10 (0.102)	0.07 (0.101)	0.13 (0.105)	0.05 (0.102)	0.05 (0.101)	0.05 (0.104)
Nonbinary	0.38 *** (0.127)	0.40 ** (0.125)	0.44 *** (0.127)	0.37 ** (0.127)	0.40 ** (0.124)	0.44 *** (0.127)
Age	0.02 (0.039)	0.02 (0.038)	0.01 (0.039)	0.03 (0.039)	0.03 (0.038)	0.01 (0.039)
Race/ethnicity (white is reference)						
Bi/Multiracial	0.23 * (0.114)	0.30 ** (0.114)	0.27 * (0.116)	0.08 (0.114)	0.10 (0.113)	0.05 (0.115)
Latine	0.13 (0.141)	0.09 (0.138)	0.04 (0.14)	0.04 (0.141)	0.03 (0.137)	−0.01 (0.14)
Asian	0.08 (0.133)	0.05 (0.129)	0.05 (0.131)	0.15 (0.133)	0.08 (0.128)	0.12 (0.13)
Black	0.09 (0.128)	0.02 (0.125)	0.03 (0.124)	−0.10 (0.128)	−0.16 (0.125)	−0.10 (0.123)
Family socioeconomic status (High SES is reference)						
Low SES	0.23 (0.132)	0.24 (0.13)	0.20 (0.13)	0.28 * (0.132)	0.29 ** (0.129)	0.20 (0.13)
Med SES	0.09 (0.092)	0.05 (0.091)	0.05 (0.092)	0.18 * (0.092)	0.14 (0.09)	0.16 (0.092)
Constant	−0.42 (0.634)	−0.49 (0.629)	−0.58 (0.642)	−0.36 (0.633)	−0.35 (0.626)	−0.08 (0.639)
Observations	555	577	556	555	577	556
Adjusted R^2^	0.064	0.049	0.051	0.062	0.052	0.061
Residual Std. Error	0.961	0.962	0.956	0.960	0.958	0.951
F Statistic	3.705 *** (df = 14; 540)	3.116 *** (df = 14; 562)	3.110 *** (df = 14; 541)	3.607 *** (df = 14; 540)	3.274 *** (df = 14; 562)	3.579 *** (df = 14; 541)

Note. All continuous variables except degree were standardized, including the outcome. Standard errors are shown in parentheses. All models included controls for gender, age, race/ethnicity, and SES. Psychological distress is measured as anxiety and depression. The scale average was used for participant sociopolitical action, critical agency, and critical reflection. The single item for all three measures of critical consciousness was used for the difference between ego and alters. The difference between ego and alters is measured as the average difference between the participant and each of their friends. That is, the difference will have positive values if the participant is higher than their friends on the attribute, whereas the difference will have negative values if the participant is lower than their friends on the attribute. Consequently, positive coefficients for the term indicate that the extent to which a participant has a higher level of the particular critical consciousness component than their friends is related to higher values of the outcome.

* *p* < 0.05;

** *p* < 0.01;

*** *p* < 0.001.
